# *Ascaridia galli* infection in chicken: Pathobiology and immunological orchestra

**DOI:** 10.1002/iid3.1001

**Published:** 2023-09-29

**Authors:** Nusrat Nowrin Shohana, Sharmin Aqter Rony, Md. Haydar Ali, Md. Shahadat Hossain, Sharmin Shahid Labony, Anita Rani Dey, Thahsin Farjana, Mohammad Zahangir Alam, Md. Abdul Alim, Anisuzzaman

**Affiliations:** ^1^ Department of Parasitology Bangladesh Agricultural University Mymensingh Bangladesh; ^2^ Department of Pathology and Parasitology, Faculty of Veterinary and Animal Science Hajee Mohammad Danesh Science and Technology University (HSTU) Dinajpur Bangladesh

**Keywords:** age, sex and breed specific immunity, Immunology, maternal immunity, pathology, *Ascaridia galli*

## Abstract

**Background:**

*Ascaridia galli* is the largest gut‐dwelling helminth of chickens, which confers adverse effects on meat and egg production; thus, on the animal protein supply and the economy. Both adult and immature parasites affect gut health, but larval stages play a major role in pathology.

**Aims:**

Here, we present immunology and pathology of *A. galli* in chickens.

**Materials and Methods:**

Literatures were surveyed through online platforms such as PubMed, Google Scholar and Researchgate.

**Results:**

The larvae cause excessive mucus production, damage to the intestinal gland, hemorrhage, anemia, diarrhea, and malnutrition. The adult worms can cause death by intestinal obstruction and intussusception. Although both cellular and humoral immunity are involved in fighting against ascariasis, the role of naturally acquired immunity is poorly defined. In cellular immunity, Th‐2 cytokines (IL‐4, IL‐5, IL‐9, and IL‐13), goblet cells (mucin), gut‐associated lymphoid tissues, CD8α+ intraepithelial cells, TCRγδ + T cells, and TGF‐β4 form a protective band. Type 2 immunity provides protection by forming a network of endogenous damage‐associated molecular patterns, chitin, and parasitic antigens. Among antibodies, IgY is the most prominent in chickens and provides temporary humoral protection. During parasitic infection, infiltration of various immune cells is evident, especially in the intestinal epithelium, lamina propria, and crypts of the duodenum and jejunum. In chickens older than 12 weeks, gradual reduction of worm burden is more successful than the younger birds. Female chickens exert a short‐lived but higher level of protection by passing IgY to chicks in the form of egg yolk antibodies. In laying conditions, immunity differs between breeds. This review provides an overview of the silent but inevitable pathological changes induced by *A. galli* and the interaction of host immunity with the parasite.

## Graphical abstract

*Ascaridia galli* is the most common helminth in chickens. It is more common in young chickens. The helminth induces huge economic losses in poultry.

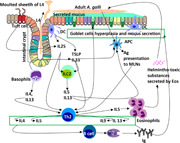

## 1 INTRODUCTION

The poultry sector, which has an extremely important place in terms of food safety and nutrition, is the fastest‐growing agricultural subsector, especially in developing countries. Poultry is raised by approximately 80% of rural households in developing countries. According to FAO estimates, the global poultry population has produced 83 million tons of eggs and 33 million tons of meat in 2021.^1^ Indigenous chickens are popularly reared in backyard or semi‐intensive systems, which is very attractive to the resource‐deprived segment of the global population, particularly by women because of minimum requirement of investment and lower food supplement. Backyard system contributes 8% of total egg production and 2% of total meat production.^2^, ^3^ The scavenging nature of the chickens facilitate easy fecal contamination of the premises, and the global warming/hot humid climatic condition provides suitable condition for parasite egg development, including the soil‐transmitted helminths. *Ascaridia galli* (Schrank, 1788), the largest nematode of chickens, is the most frequently encountered problem (up to 90%) of indigenous chickens.^4^ Ascaridiasis in chickens is highly prevalent in many countries around the globe, like Germany (88%), Sweden (77.1%), Bangladesh (61%), India (32.97%), and Tanzania (32.3%), where birds were reared in free range system compared to Ghana (30%) and Serbia (15.6%–24%), where intensive rearing system was applied.^5^, ^6^, ^7^, ^8^, ^9^, ^10^, ^11^

*Ascaridia galli* affects the small intestine, especially the duodenum of chickens, pigeons, and wild birds.^12^, ^13^, ^14^ The parasite can be associated with anemia, emaciation, and reduction in production efficiency.^15^, ^16^ Direct losses are caused by obstruction and damage to the intestinal tract, resulting in malabsorption and malnutrition, alteration of beneficial gut microflora, immunosuppression, and increased susceptibility to concurrent infections.^17^ In addition, *A. galli* can act as a vector for other pathogens such as bacteria (e.g., *Salmonella enterica*)^18^ and can impair the humoral immune responses (HIRs) following vaccination against other pathogens (e.g., Newcastle disease virus).^19^ Concurrent infection with *A. galli* and *Escherichia coli* ^20^ or *Pasteurella multocida* ^21^ has been shown to have significant effects on weight gain and egg production. Because the life cycle of *A. galli* is direct, transmission of the parasite is very rapid, especially in deep litter systems. In addition, earthworms act as transport hosts and may play a critical role in the transmission cycle in rural scavenging and semi‐scavenging settings.^22^, ^23^

The intestinal epithelium acts as a communication network for this gut‐dwelling nematode as it transmits signals to the immune systems (innate and acquired) in the underlying mucosa.^24^ Macroscopically, *A. galli* infection includes a thickened intestinal wall with hemorrhagic spots along with edema and infiltration of lymphoid cells mixed with eosinophils.^25^ Occasionally, ulcerative proventriculitis can also be detected in ascaridiasis.^26^ The immunological consequences are the alteration in the number and activity of Th2 lymphocytes, Toll‐like receptor expression, the release of the endogenous damage‐associated molecular pattern (eDAMP) or alarmins, and immunomodulatory proteins.^27^, ^28^ Since an anthelmintic vaccine against *A. galli* is yet to be developed and commercialized, an anthelmintic‐based control program is the main tool against this helminth.^29^ Piperazine and levamisole have been used against *A. galli* for decades.^17^ Although anthelmintic resistance (AR) against *A. galli* is yet to be reported, however, AR to other helminths is well documented.^30^, ^31^ In addition, recently, we found that piperazine has very limited activity against several gastro‐intestinal tract dwelling helminths, including *A. galli* (unpublished data). While the concept of AR is one of the burning issues around the world, the use of plant materials with anthelmintic activity like papaya seed, papaya latex, neem seed, and leaf extract of pine apple could be an easier and cheaper escape route.^32^, ^33^ Therefore, a thorough, up‐to‐date understanding of the biology, pathology, and immunology of *A. galli* is needed to develop a sustainable control strategy to mitigate the problem and make poultry production economically viable. This review focuses on the pathobiology and immunity induced against *A. galli* infection in chickens.

## 2 TRANSMISSION AND PATHOBIOLOGY

**Figure 1:**
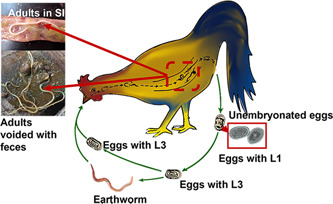
Schematic presentation of the life cycle of *Ascaridia galli*. L, larva; SI, small intestine.

*Ascridia galli* leads a direct life cycle (Figure 1) involving a single host. The sexually mature adult worms live in the small intestine, lay eggs, and are expelled into the external environment. The oval eggs are enveloped with three layers, namely, the vitelline membrane (the inner permeable layer), a thick, resistant covering, and a thin albuminous layer,^34^ which make them resistant to desiccation and allow long survival in the external environment. Eggs do not hatch in the environment; rather develop larvae within the egg, molt continuously, and eventually become the third larval stage (L3). An egg with L3 is the infective stage. During development, eggs begin to divide into the two‐cell stage within 24 h and continue to develop into the three‐cell stage within 48 h and the four‐cell stage within 72 h. This fourth stage develops within eggs, known as morula with blastomeres. A fully mature infectious L3 stage is formed after 11–12 days.^35^ Transmission occurs through ingestion of contaminated food/water or mechanically by earthworms.^13^, ^34^ Earthworms act as paratenic hosts that ingest the infectious stage and become infectious to the definitive hosts. Infection by ingestion of earthworms is easier than picking up eggs in nature. However, the earthworm must be consumed within 96 h to result in effective transmission of *A. galli*; otherwise, the infective stage may be excreted with the gut contents of the annelids, or the eggs can be hatched in the earthworm intestine.^23^ The stimulating factors such as temperature, pH, and carbon dioxide levels initiate egg hatching and release L3 within 24 h of infection,^36^ in the anterior portion of the jejunum, the first predilection site of newly hatched larvae.^37^ The life cycle of *A. galli* includes both free‐living larvae and parasitic stages (infective eggs to adult worms). Both stages are responsible for pathological sequelae^38^, ^39^ and adopt sequentially through the mucosal and histotrophic stages.^40^ In necropsy, bundles of parasites are usually seen, indicating a massive dose of infection.

During the first week of infection, most larvae settle in the anterior half of the jejunoileal section but migrate posteriorly as infection progresses. Thus, one subpopulation of larvae, which inhabit mainly in the lumen, grows over time, while another subpopulation remains dwarfed and attached with the mucosa. Later, both subpopulations migrate to a posterior localization in the gastro‐intestinal tract.^41^

The next larval stage is histotrophic, which can last 3–54 days before the larvae move to a final stage in the lumen. Most larvae (63%) are found in the lumen in contact with the epithelium in the crypts of Lieberkühn after 3 days of infection, and 37% of larvae are found in the tunica mucosa.^40^ This study also suggested that the highest number of larvae remained in the crypts (51%), followed by the transitional zone (31%) and the villous zone (18%).^40^ The duration of this phase is usually dose‐dependent. When the histotrophic phase ends, the larvae return to the lumen of the intestine and become adults. After maturation, adult female worms produce a large number of eggs. The prepatent period is about 4–8 weeks,^42^ depending on the age of the host. During the so‐called mucosal or histotrophic phase, when large numbers of larvae invade the duodenal mucosa, they can cause hemorrhagic enteritis by destroying the intestinal epithelium, leading to malabsorption and eventually malnutrition.^43^ Ascaridiasis is often associated with anemia, decreased sugar levels, and necrosis of the mucosa, resulting in diarrhea, anorexia, weakness, ruffled feathers, and a dirty vent.^26^, ^44^ The pathogenesis of *A. galli* in chickens is mostly associated with hypertrophy of the intestinal villi infiltration of inflammatory cells, especially eosinophils, lymphocytes, and macrophages. Necrosis of the crypts of Lieberkühn is associated with the histotrophic phase of larvae.^24^ Infection can result in poor growth followed by poor performance.^45^ Infection with large numbers of adult worms results in obstruction of the small intestinal lumen and also intussusception of the intestine due to hypermotility leading to death.^45^ Sometimes, *A. galli* toxins are released with the secretory and excretory (E/S) products of the worms, which impair the enzymatic process of the intestine and further impede the absorption of nutrients through the intestinal wall.^46^

## 3 IMMUNITY

The defense mechanism of the body depends on both cellular and humoral components. In poultry, blood parameters (e.g., acute phase proteins) and intestinal lymphocyte subpopulations such as innate lymphoid cells are greatly modulated by helminth infections.^47^ Blood parameters like mannose‐binding lectin and alpha 1‐acid glycoprotein can even be used as indicators for early disease diagnosis,^48^ which can be very useful for monitoring the health status of poultry. Worm expulsion was closely related to the developmental stage of the worms, with the elimination of juvenile stages being particularly high. A very small percentage of the worms are nevertheless able to survive and reach sexual maturity.^41^

## 4 CELLULAR IMMUNITY AGAINST *A. galli*

The endogenous immunity of chickens counters both intracellular (e.g., bacteria) and extracellular (e.g., nematodes) invaders mainly through Th1‐ and Th2‐type immune responses, respectively.^17^, ^49^ The collagen‐based cuticle with carbohydrate‐rich surface coatings of the parasites, as well as their ability to change antigenic surfaces through multiple molts during the development cycle,^50^ play a critical role in how the parasitic worms evade the host's innate immune system.^51^, ^52^ There are a few studies describing naturally acquired immunity to *A. galli*.^45^ Although specific information on *A. galli* is not known, it is generally assumed for other gastrointestinal nematodes that the Type 2 response includes several biological processes that serve to disrupt the parasite niche in the gut by strengthening the physical barrier and promoting tissue repair.^53^ These mechanisms are highly coordinated and involve several different cell types and effector molecules that have been implicated at various stages of response.^30^, ^54^ Given this, intestinal nematodes tend to cause more tissue destruction than other pathogens due to their body size and invasiveness. It is perhaps more plausible that Type 2 immunity results from the combined recognition of eDAMP or alarmins and worm‐derived molecules (including chitin) that become available for uptake after larval molting. In addition, parasite‐derived antigens that are continuously secreted during infection also elicit an immune response.^54^ Pattern recognition receptors are constitutively expressed at key ports (e.g., skin, lungs, and intestinal epithelia) of pathogens and have been shown to interact with pathogen‐associated molecular patterns (PAMPs) or eDAMP during the progression of various parasitic infections, including helminths and others.^55^, ^56^

CD4+ Th2‐mediated immunity is characterized by secretion of Type 2 signature cytokines (IL‐4, IL‐5, IL‐9, and IL‐13), activation of alternatively activated macrophages, eosinophils, basophils, and mast cells.^56^, ^57^ Such a response to enteric nematodes, including *A. galli*, is associated with hyperplasia of goblet cells, increased numbers and degranulation of mast cells,^58^ and an increase in heterophils.^40^ Goblet cells are the cells of the innate immune system that are the main source of mucins. Mucins are the main macromolecules of the intestinal mucus barrier.^57^ The enlargement of goblet cells leads to increased secretion of mucins. The mucus restricts the movement of the worms by covering their cuticle; thus, it hampers the attachment of worms to the intestinal mucosa, and the worms are eventually removed from the intestinal lumen with the feces.^59^, ^60^ Granulocytes, especially eosinophils, release toxic substances that are considered effective against extracellular organisms like nematodes.^61^ In addition, the gut mucosal immune system is a compartmentalized part of the immune system that provides local immunity because it possesses secondary lymphoid tissues (T, B, and dendritic cells). Once mucosal immune cells are stimulated by luminal antigens, they infiltrate into the diffuse areas of mucosal tissue (e.g., the lamina propria of intestinal villi) and exhibit immune effector functions.^62^ Gut‐associated lymphoid tissues (GALTs) are well‐developed in birds. It consists of lymphoid cells located in the epithelial lining and the lamina propria, as well as specialized lymphoid structures such as Peyer's patches and cecal tonsils.^63^

The follicle‐associated epithelium covering the GALTs has specialized microfold cells that actively internalize luminal antigens to trigger antigen‐specific immunoglobulins, such as IgA production.^64^, ^65^ Chickens exposed to nematodes induce polarization of Th1/Th2 immune responses.^17^, ^66^ Immunity to *A. galli* is highly gut‐specific.^40^, ^44^, ^49^ Coordinated and measured involvement of different subpopulations of immune cells in the different layers of the intestinal wall, particularly in the epithelium, lamina propria, and crypts of duodenum and jejunum, was elegantly demonstrated.^44^ It was also described that the population of CD8α+ intraepithelial lymphocytes (CD8α+ IEL) in the crypts of the jejunum decreased significantly 2–3 weeks after infection. Another study reported that the number of CD4+ IEL in the duodenum and jejunum infected with *A. galli* increased twofold to threefold 1‐week postinfection (pi). However, the relative number of CD8α+ IEL decreased in the duodenum and jejunum 2‐week pi.^67^ TCRγδ+ T cells were also shown to increase (∼45%) in *A. galli* infections within 1‐week pi in the duodenum. However, no changes were observed in IgA + B lymphocytes in the jejunum (2‐week pi) and duodenum (1‐ and 2‐week pi) (Table 1).

**Table 1:** Reports on cellular and humoral immunity against *Ascaridia galli* infection in chickens.

Types of immunity	Source
*A. galli* infection develops both cellular (T helper type 2/Th2 immune response) and humoral (IgY antibody‐mediated) immunity. However, elevated IgY level does not confer permanent protection against *A. galli*.	[17, 66, 67, 68, 69, 70]
Immunity to *A. galli* is highly gut‐specific.	[49, 69]
Immune cells are predominantly found in duodenum and jejunum.	[44]
In CD4+ Th2‐mediated immunity, IL‐4, IL‐5, IL‐9, and IL‐13 are involved.	[57, 71, 72]
*A. galli* induced goblet cell hyperplasia, increased number of heterophils and mast cells.	[73]
CD4+ intraepithelial lymphocytes are increased two to three times in infected duodenum and jejunum at 1‐week postinfection.	[44]
IFN‐γ, IL‐1β, IL‐2β, and IL‐18 from splenic cells increased at 6‐week postinfection.	[66]
DEFβ1 (Beta‐defensin 1) significantly declined at 2 weeks postinfection but increased after 6 and 8 weeks.	[74]
TCRγδ+ T cells in the duodenum are increased within 1‐week postinfection. Worm burden is associated with the influx of both αβ, including CD4+ cells and γδ T cells in the jejunal mucosa.	[44]
Mannose‐binding protein (MBP) significantly increased in splenic tissues.	[74]
Jejunal mast cells result in the degradation of the *A. galli* cuticle.	[74]
Humoral immune response can only impair larval growth.	[42, 70]
IL‐13 activates the STAT6 pathway in *A. galli* infection, resulting expulsion of worms.	[19, 44, 75]
Th2 cytokines associated with short histotrophic phase of *A. galli*.	[49, 76]
*A. galli* larvae control the antibody production level in both plasma and egg yolk.	[77]

Upon infection with *A. galli*, Th2 cytokines, interleukin IL‐4, and IL‐13 are upregulated in the spleen and ileal tissue.^71^ In addition, splenocytes from chickens infected with *A. galli* showed increased expression of IFN‐ƴ, IL‐1β, IL‐12β, and IL‐18 at Week 6 pi, but not at Week 2 or Week 9 pi, except for IL‐8.^66^ Expression of IL‐8 was upregulated at both Week 2 and Week 6 pi. On the other hand, DEFβ1 (beta‐defensin 1) expression significantly decreased at 2‐week pi and increased at Weeks 6 and 8 pi. Acute phase proteins, such as mannose‐binding protein, are significantly increased in the spleen tissue of chickens infected with *A. galli*.^66^ Increased expression of TGF‐β4 was also observed; however, IL‐10 was not increased but rather decreased.^71^ The Th2 signature cytokine IL‐13 was upregulated in the spleens of chickens infected with *A. galli* at 2 weeks pi but not at later stages of infection^66^ (Table 1). It remains to be determined whether the proinflammatory responses are caused by *A. galli*‐specific PAMPs, host‐specific DAMPs released by tissue damage, or the DAMP homologues secreted by the parasites or opportunistic secondary microbial pathogens. In the jejunal mucosa, there is an influx of both αβ, including CD4+ cells and ƴδ T cells associated with worm burden, and the highest worm burden results in the highest mRNA expression.^44^

## 5 HUMORAL IMMUNITY AGAINST *A. galli*

Chickens infected with *A. galli* showed upregulation of mRNA of the cytokines IL‐4 and IL‐13 in spleen and ileal tissue^14^, ^44^, ^71^ and developed a systemic HIR.^68^, ^69^ Other research has shown that birds infected with *A. galli* develop both cellular (T2‐type) and humoral (IgY antibodies, referred to as IgG) immune responses.^70^ Chicken sera contain three different types of antibodies, such as IgA, IgM, and IgY. Considering the structure, molecular weight, and immunoelectrophoretic mobility, chicken IgA and IgM are similar to mammalian IgA and IgM,^78^ and IgY is the most dominant antibody in birds, reptiles, amphibians, and lungfish.^79^ Elevated levels of IgY level have been found in *A. galli* infection, but this does not provide any permanent protection.^67^, ^69^, ^70^ In fact, the HIR does not provide adequate protection against this nematode and, therefore, cannot prevent reinfection. It can only affect larval growth rather than a complete elimination from the host.^42^, ^70^ The onset of HIR occurs within 2 weeks of infection with *A. galli*, but serum titers do not correlate with worm burden or fecundity of the worm.^37^, ^70^ However, the number of *A. galli* larvae count correlates significantly with IgY levels at 2 weeks pi.^49^ The delayed trend of HIR and the lower IgY level might be related to the age of the bird.^49^

**Figure 2:**
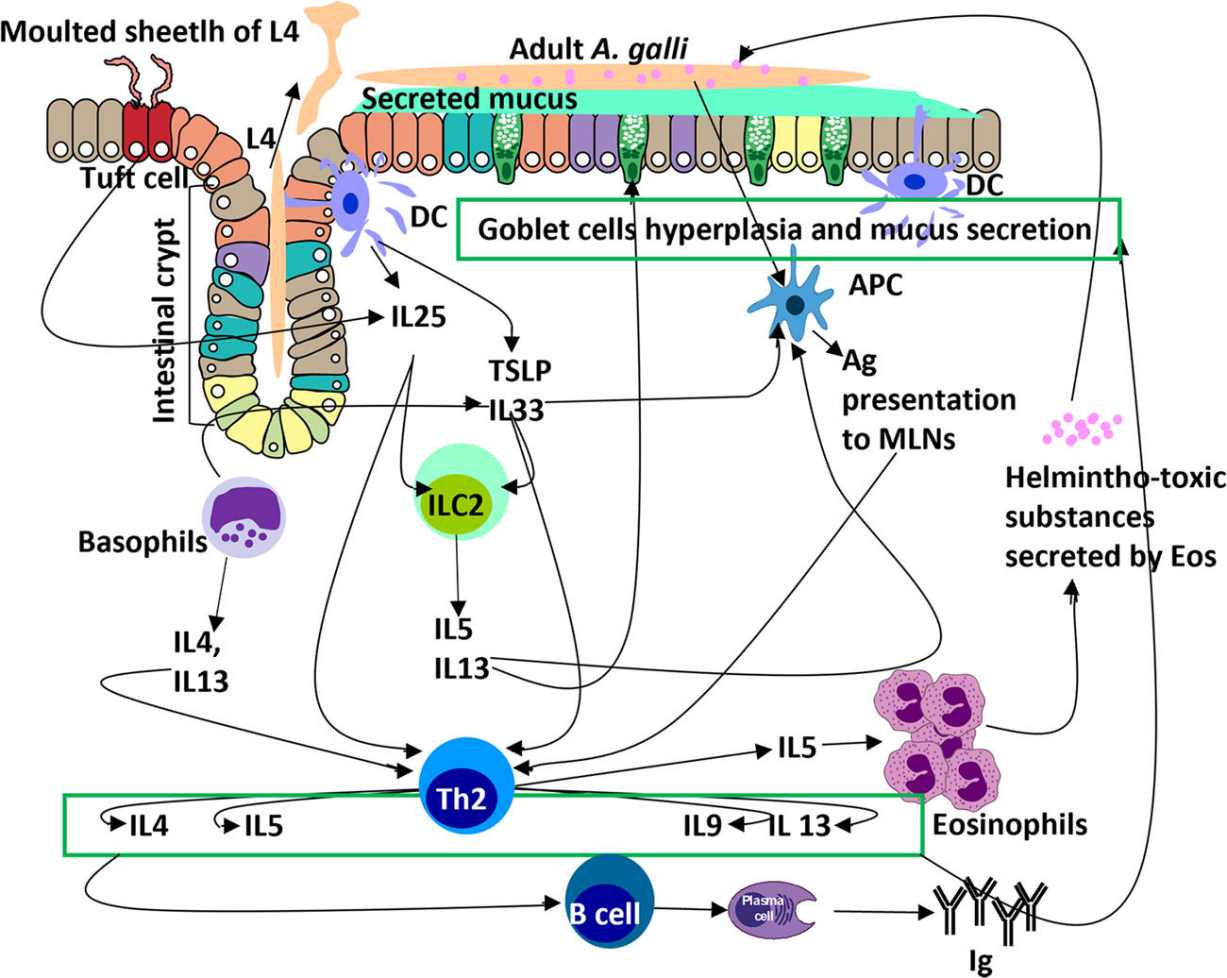
Schematic presentation of immunological orchestra elicited against *Ascaridia galli* infection. Ag, antigen; APC, antigen‐presenting cells; DC, dendritic cells; Eos, eosinophils; ILC, innate lymphoid cells; IL, interleukin; MLNs, mesenteric lymph nodes; TSLP, thymic stromal lymphopoietin.

Both humoral and local immune responses are associated with worm expulsion, with the latter likely to have strong effects.^49^ There are three distinct phases of *A. galli* expulsion: (1) The first phase, which depends on larval hatchability and the transit time of the host's digesta, allows effective larval establishment on the first day of infection. (2) The second phase, which is the most efficient. It is partially species‐specific and acts on both tissue‐associated larvae and juvenile stages located in the lumen by activating humoral and cell‐mediated immunity (Table 1). Targeted expulsion of the first‐generation worms, slow‐growing larvae, and posterior localization are more evident in this phase. (3) In the third phase, only a few *A. galli* are expelled.^49^ An immunological orchestra elicited against *A. galli* infection is depicted in Figure 2.

The short‐term upregulation of Th2 cytokines may be related to the short histotrophic phase of *A. galli*, which is an obligatory component of the early infection stage.^49^ This is consistent with larval‐dependent antibody production.^77^ In fact, the number of larvae, rather than the number of mature worms, influences levels of antibodies in both plasma and egg yolk. The antibody titers might be influenced by several factors, such as the antigen type and dose, the used adjuvant, the route of application, the inoculation frequency, age, and stage of development in birds.^80^

## 6 AGE‐RELATED IMMUNITY AGAINST *A. galli*

A plethora of literatures suggest that there is age‐dependent immunity.^81^, ^82^, ^83^, ^84^ Older chickens are more able to resist infection with *A. galli* than younger birds.^83^ In birds aged >3 months, the histotrophic phase is much longer than in younger birds (≤3 months); therefore, worms mature more rapidly in younger birds and affect the prepatent period.^13^ In the Lohmann LSL layer, age does not count as a significant determinant of resistance against *A. galli*, but a bird's hormonal and immune status does.^84^ In another study, high levels of growth inhibitory factors in older birds were found to prevent the development of *A. galli* infection.^85^ Interestingly, age does not ensure protection in layers (Table 2). Laying hens are more susceptible to this nematode infection due to hormonal changes and lower antibody levels, making them immunocompromised.^84^

**Table 2:** Reports on age, sex‐, and breed‐related immunity against *Ascaridia galli* infection in chickens.

	Source
Age‐related immunity	
Adult chickens (>3 months) can prevent *A. galli* infection more strongly than younger birds.	[12, 86]
In young birds, the prepatent period is short because of the shorter histotrophic phase	[86]
Laying hens are more susceptible to *A. galli* infection due to alteration of hormone level	[85]
High levels of growth‐inhibiting factors in older birds resist the infection.	[86]
Sex‐related immunity	
Female chicks have higher levels of antibody than male due to *A. galli* infection, but it is not protective enough.	[87, 88]
Alteration in protective immune responses (humoral and cellular) greatly hampers vaccine‐induced immunity in laying hens.	[19]
Breed‐related immunity	
Susceptibility to *A. galli* infection differs genetically in different layer‐lines like Danish Landrace and Lohman Brown.	[89]

## 7 SEX‐ AND BREED‐RELATED IMMUNITY AGAINST *A. galli*

*A. galli*‐specific serum antibodies develop to a greater extent in female chicks than in males, independent of dam/offspring infection levels. As chickens become adults, the population of T lymphocytes decreases.^52^, ^87^, ^88^ On the other hand, infection with *A. galli* can alter protective immune responses (humoral and cellular), thereby severely compromising vaccine‐induced immunity in laying hens.^19^ The major histocompatibility complex or B‐complex in chickens is associated with resistance or susceptibility to disease at the individual level.^90^, ^91^ Susceptibility to infection with *A. galli* differs genetically in different lines of layers. The Danish Landrace (DL) was found to have a higher worm burden and egg count than the Lohmann Brown (LB) (Table 2) breed of chickens. Both DL and LB showed a self‐cure mechanism, that is, elimination of adult parasites at the time of infection with infectious stages, which is a well‐recognized phenomenon in sheep indicated against *Haemonchus contortus*.^91^

## 8 MATERNAL IMMUNITY AGAINST *A. galli*

Maternal immunity, a natural passive immunity, is conferred by the administration of preformed immunoglobulins (Igs) and provides immediate but short‐lived protection against any infection.^92^, ^93^ Studies suggest that maternal antibodies are the main escape route for very young chicks.^94^, ^95^ IgG (IgY) transferred from the hen can ensure an effective immune response in the young chicks. IgY accumulates predominantly in the egg yolk (5–15 mg/mL), while IgA (0.3–0.5 mg/mL) and IgM (1–3 mg/mL) are found in egg whites.^96^ Egg yolk, ovarian follicles, yolk sac membranes, and oviductal secretions are the main repositories for transferring maternal IgY to hatchlings.^68^, ^97^ The yolk sac membrane has specific receptors for IgY,^98^ and the amount of IgY transferred to the yolk is directly proportional to serum antibodies.^94^ Studies have confirmed that there is a strong correlation between plasma and egg yolk IgY levels in hens infected with *A. galli*.^52^, ^94^ The production of *A. galli*‐specific Ig in plasma and egg yolk is dependent on the infective dose and the duration of infection. Plasma antibody (PAB) is induced much earlier than egg yolk antibody (EAB); therefore, PAB is better for providing evidence of early infection.^77^ However, both PAB and EAB only indicate the infection dynamics of the parasite, not protection against the worm (Table 3). The number of antibodies present in the egg yolk correlates strongly with the antibody levels of the hens. Nevertheless, maternal antibodies can provide some protection to the chicks, but they do not ensure permanent protective immunity against infection with *A. galli*.^52^

**Table 3:** Reports on maternal immunity against *Ascaridia galli* infection in chickens.

Maternal immunity	Source
Egg yolk, ovarian follicles, yolk sac membranes, and oviduct are the major sites to transfer maternal IgY to hatchlings.	[68, 97, 99]
IgY is predominantly found in egg yolk (5–15 mg/mL).	[96, 100]
*Ascaridia galli*‐specific antibodies production in plasma and egg yolk is dependent on the infective dose and duration of infection.	[77]
Plasma and egg yolk antibodies indicate only infection but do not give any protection against the worm.	[52]
Plasma antibody is more suitable to get hints regarding early infection.	[77]
Maternal immunity exerts immediate but short‐lived protection.	[92]

## 9 CONCLUSIONS

Ascariasis is a highly prevalent helminth infection in chickens reared in scavenging and semi‐scavenging settings. It mainly affects young birds but is not infrequent in adults as well. Until recently, there is no effective vaccine, and anthelmintics are the main means of tackling the problem. As the prepatent period of *A. galli* is 4–8 weeks, birds can be treated with an effective anthelmintic (e.g., levamisole) at 4‐ to 6‐week intervals. Humoral immunity does not provide protection; therefore, fostering research activities targeting cell‐mediated immunity is essential. The development or detection of resistant breeds or strains and their dissemination among farmers will help to alleviate the problem. The identification of gene(s) associated with the helminth resistance phenomenon and a controlled breeding policy to develop resistant poultry strain(s) would be another way to reduce the losses associated with *A. galli* in the poultry industry. Besides, proper nutrition of poultry and the use of botanicals with anthelmintic activity could be revolutionary approaches for sustainable control of *A. galli*.

## AUTHOR CONTRIBUTIONS

*Methodology, writing original draft, revision*: Nusrat Nowrin Shohana, Sharmin Aqter Rony, and Md. Haydar Ali. *Methodology, writing original draft, revision, and editing*: Md. Shahadat Hossain, Sharmin Shahid Labony, Anita Rani Dey, and Thahsin Farjana. *Conceptualization, writing review, editing, and revision*: Anisuzzaman, Md. Abdul Alim, and Mohammad Zahangir Alam. All authors have read and approved the final manuscript.
